# Salivary gland-sparing other than parotid-sparing in definitive head-and-neck intensity-modulated radiotherapy does not seem to jeopardize local control

**DOI:** 10.1186/1748-717X-8-132

**Published:** 2013-05-30

**Authors:** Enrique Chajon, Caroline Lafond, Guillaume Louvel, Joël Castelli, Danièle Williaume, Olivier Henry, Franck Jégoux, Elodie Vauléon, Jean-Pierre Manens, Elisabeth Le Prisé, Renaud de Crevoisier

**Affiliations:** 1 Centre Eugène Marquis, rue de la bataille Flandres Dunkerque, CS44229, Rennes, Cedex 35042, France; 2 INSERM, Rennes, U1099, France; 3 Université de Rennes 1, LTSI, Rennes, France; 4 Centre Hospitalier Régional Universitaire Pontchaillou, Rennes, 35000, France

**Keywords:** Head -and-neck cancer, IMRT, Xerostomia, Salivary glands

## Abstract

**Background:**

The objective was to analyze locoregional (LR) failure patterns in patients with head-and-neck cancer (HNC) treated using intensity-modulated radiotherapy (IMRT) with whole salivary gland-sparing: parotid (PG), submandibular (SMG), and accessory salivary glands represented by the oral cavity (OC).

**Methods:**

Seventy consecutive patients with Stage I-II (23%) or III/IV (77%) HNC treated by definitive IMRT were included. For all LR failure patients, the FDG-PET and CT scans documenting recurrence were rigidly registered to the initial treatment planning CT. Failure volumes (Vf) were delineated based on clinical, radiological, and histological data. The percentage of Vf covered by 95% of the prescription isodose (Vf-V95) was analyzed. Failures were classified as “in-field” if Vf–V95 ≥ 95%, “marginal” if 20% < Vf-V95 < 95%, and “out-of-field” if Vf-V95 ≤20%. Correlation between Vf-V95 and mean doses (Dmean) in the PG, SMG, and OC was assessed using Spearman’s rank-order correlation test. The salivary gland dose impact on the LR recurrence risk was assessed by Cox analysis.

**Results:**

The median follow-up was 20 months (6–35). Contralateral and ipsilateral PGs were spared in 98% and 54% of patients, respectively, and contralateral and ipsilateral SMG in 26% and 7%, respectively. The OC was spared to a dose ≤40 Gy in 26 patients (37%). The 2-year LR control rate was 76.5%. One recurrence was “marginal”, and 12 were “in-field”. No recurrence was observed in vicinity of spared structures. Vf-V95 was not significantly correlated with Dmean in PG, SMG, and OC. The LR recurrence risk was not increased by lower Dmean in the salivary glands, but by T (p = 0.04) and N stages (p = 0.03).

**Conclusion:**

Over 92% of LR failures occurred “in-field” within the high dose region when using IMRT with a whole salivary gland-sparing strategy. Sparing SMG and OC in addition to PG thus appears a safe strategy.

## Background

Xerostomia is one of the most common and disabling adverse effects of radiotherapy (RT) for head-and-neck cancer (HNC), inducing difficulties in swallowing and speaking, loss of taste, and dental caries, with a direct impact on patient quality of life [1]. Reducing xerostomia is a main objective of intensity-modulated RT (IMRT) in HNC. Currently, a modest but significant improvement in salivary flux parameters and subjective xerostomia has been confirmed in randomized trials, with only parotid gland-sparing [2-4].

A potential reduction in the sensation of mouth dryness has been hypothesized provided that other tissues such as submandibular (SMG) and accessory salivary glands (represented by the oral cavity [OC] volume) are spared [5]. A dose-effect relationship of mouth dryness was demonstrated for SMG with an exponential reduction in salivary output as mean dose increases beyond a threshold of 39Gy [6]. Moreover, significantly superior unstimulated salivary flow and xerostomia grades were correlated in prospective studies with contro-lateral SMG-sparing [7]. However, the SMG and OC are usually in close relationship with planning target volumes (PTV) as are the oropharyngeal mucosa and the Level II lymph nodes. Consequently, there is a risk of under-dosing these target volumes, which would increase locoregional (LR) recurrences. In contrast to parotid gland (PG)-sparing IMRT, safety data with SMG- and OC-sparing is scant. Saarilahti K et al. [8], reported that SMG-sparing using IMRT is feasible in selected patients, with no cancer recurrences observed at the vicinity of spared SMG. A recent update of this series involving a larger cohort of patients confirmed the initial results [9].

This report aimed to analyze the LR failure patterns in HNC patients treated with definitive IMRT using a comprehensive approach for salivary gland-sparing, which integrated in the planning process not only PG but also SMG and accessory salivary glands represented by OC, when definitive bilateral neck RT was indicated.

## Methods

### Patients

Between September 2009 and August 2011, 111 consecutive patients with histologically confirmed squamous cell carcinoma of head and neck were treated with IMRT at the Eugene Marquis Cancer Center radiotherapy department (Rennes, France). Overall, 41 patients who received postoperative IMRT or unilateral neck irradiation were excluded from analysis. The remaining 70 who received definitive and bilateral neck IMRT were analyzed.

Of the 70 patients, 54 exhibited locally-advanced AJCC (American Joint Committee on Cancer) Stage (III-IV) disease and 16 early-stage (I-II) disease. The patient clinical characteristics are summarized in Table 1. Staging evaluation included complete history and physical examination, appropriate endoscopic examination under anesthesia, which consisted of biopsy with detailed disease mapping and contrast-enhanced computed tomography (CT) scan of head, neck, and thorax. The 18-fluorodeoxyglucose (FDG) positron emission tomography (PET)-CT scans were obtained before treatment start, 3 months after the end of RT, and in case of suspected recurrence.

**Table 1:** **Demographic**, **tumor and treatment characteristics of the 70 patients**

***Characteristic***	***N (%)***
Age (y)	
Median	61
Range	41-91
Gender	
Male	62
Female	8
Primary site	
Oral cavity	8
Oropharynx	31
Hypopharynx	17
Larynx	10
Nasopharynx	3
Unknown primary	1
Tumor stage	
T0	1
T1	5
T2	22
T3	25
T4	17
Nodal stage	
N0	18
N1	16
N2a	3
N2b	20
N2c	8
N3	5
Overall stage	
I	1
II	15
III	9
IV	45
Radiotherapy	
Step and Shoot IMRT	65
Volumetric Modulated Arc Therapy	5
Chemotherapy	
Concurrent	
Cisplatin	22
Carboplatin/5-Fu	7
Cetuximab	12
Induction + concurrent	7
No chemotherapy	22

### Radiotherapy

All patients received definitive external beam RT using step and shoot IMRT or volumetric modulated arc therapy (VMAT). Patients were immobilized in supine position with custom aquaplast masks holding both neck and shoulders. CT contrast-enhanced images indexed every 2-3 mm were acquired extending from the vertex to the carina (Brilliance, Big Bore, Philips, Netherlands). Target and organs at risk (OAR) volumes were delineated slice by slice on CT images. The gross tumor volume (GTV) was defined by clinical examination, computed tomography, and FDG-PET scan. The GTV encompassed all visible and palpable primary and nodal diseases. The high-risk clinical target volume (CTV) was defined by GTV plus areas potentially containing microscopic disease limited by anatomic barriers or by GTV plus 10 mm margin when an anatomic barrier was not clearly identified (ex: tongue base), or when the lymph node levels containing involved lymph nodes and neighboring node levels were considered at risk of subclinical involvement greater than 15-20% [10]. The low-risk CTV encompassed the remaining lymph node areas considered at low-risk (greater than 5%) for potential microscopic spreading [11,12]. The planning target volume (PTV), aimed to account for setup uncertainties, was defined using an additional margin of 5 mm around the GTV (PTV66 or 70), the high-risk CTV (PTV60 or 63), and the low-risk CTV (PTV54 or 56).

Patients with Stage I-II disease were treated with a simultaneously modulated accelerated RT (SMART) technique: The dose prescribed was 66Gy in 2.2Gy daily fractions over 30 days to the PTV66; 60Gy in 2Gy daily fractions to the PTV60; and 54Gy in 1.8Gy daily fractions to the PTV54 [13].

Patients with locally-advanced disease (Stage III-IV) were treated with a simultaneously integrated boost (SIB) technique: The dose prescribed was 70Gy in 2Gy daily fractions over 35 days to the PTV70; 63Gy in 1.8Gy daily fractions to the PTV63; and 56Gy in 1.6Gy daily fractions to the PTV56 [11].

OAR included the spinal cord, brainstem, mandible, larynx, bilateral inner ears, esophagus, pharyngeal constrictors, OC, parotid glands, and SMG. In nasopharyngeal, oropharyngeal or laryngeal cancer cases, selective Level IB-sparing was considered in the N0 neck or if Level II was not grossly involved (no more than 1–2 lymph nodes of no more than 3 cm in diameter). In patients with well-lateralized primary OC (gingivobuccal mucosa or alveolar ridge) tumors and early stage, selective controlateral SMG-sparing was also considered.

### Dosimetric considerations

Treatment planning was carried out on the Pinnacle (Version 9.0) treatment planning system, using 6MV photon beams from an Elekta Synergy linear accelerator. The highest priority was assigned to PTVs with the following constraints: more than 95% of any PTV should receive more than 95% of the prescribed dose or more than 98% of any PTV should receive more than 90% of the prescribed dose; no more than 20% of any PTV could receive more than 110% of the prescribed dose; no more than 1% or 1 cm^3^ of the tissue outside the PTV should receive more than 110% of the dose prescribed to the primary dose target (PTV 70 or 66).

The following OAR dose constraints were used [14]: brainstem and spinal cord maximum doses (D2%) were 50Gy and 45Gy, respectively; for the parotid glands, mean dose (D *mean*) was <26Gy or the maximum dose received by 50% of the volume was <30Gy; for the SMG, D *mean* was ≤39Gy; for OC (defined as previously described by Eisbruch et al. [5]), D *mean* was as low as possible; for the larynx, D *mean* was ≤45 Gy; for the pharyngeal constrictors, D *mean* was ≤40 Gy; for the esophagus, D *mean* was ≤35 Gy; for the inner ears, D *mean* was ≤40Gy; for the mandible, D2% was ≤65Gy.

### Definition and dosimetric analysis of failure volumes (Vf)

**Figure 1:**
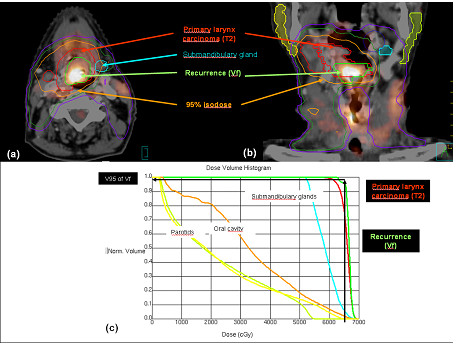
**Example of in**-**field recurrence. **The planning CT has been registered with the FDG-PET-CT imaging of the recurrence. The failure volume (*Vf) *appears fully within the 95% PTV isodose (**a **and **b**). The *Vf*-V95 is equal to 99.9% (**c**).

**Figure 2:**
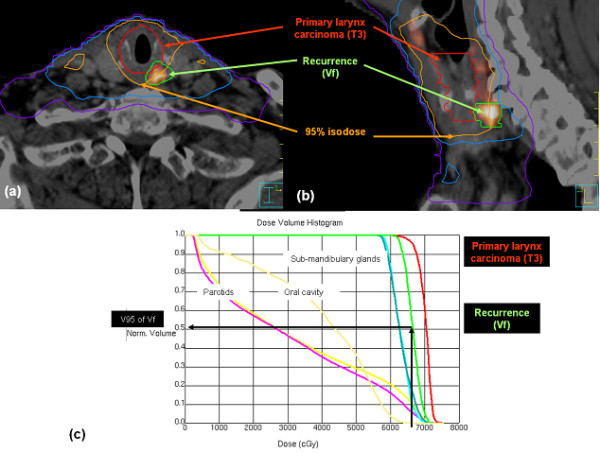
**Example of marginal recurrence. **The planning CT has been registered with the FDG-PET-CT imaging of the recurrence. The failure volume (*Vf) *appears partially outside the 95% PTV isodose (**a **and **b**). The *Vf*-V95 is equal to 50% (**c**).

For all patients with local or regional failure, FDG-PET and CT scans of the recurrence were rigidly registered to the initial treatment planning CT data set using Pinnacle Version 9.2. Failure volumes (*Vf*) were defined and delineated taking into account clinical, radiological, and histological data. Dose-volume histograms (DVH) were calculated in order to analyze the dose of irradiation received by *Vf*. Failures were classified as “in-field” if >95% of *Vf* was covered by 95% of the prescription isodose (as shown in Figure 1), as “marginal” if 20%-95% of *Vf* was encompassed by 95% of the prescription isodose (as shown Figure 2), and as “out-of-field” if <20% was contained within the 95% of corresponding PTV isodose line [15].

### Statistical analysis

Cumulative incidence of LR failure and overall survival (OS) were estimated using the Kaplan-Meier method. The relapse time to treatment failure was calculated using the first RT day as the starting point. Persistent disease was defined if tumor presence was proven within 6 months following RT completion. Disease recurrence was defined as tumor appearing more than 6 months after therapy completion. Correlation between *Vf*-V95 and mean doses in salivary glands (PG, SMG, and OC) was assessed using Spearman test. The impact of dose parameters on LR recurrence risk was assessed by Cox analysis.

All analyses were performed using Statistical Package for the Social Sciences, Version 10.0 (SPSS, Chicago IL).

## Results

### Failure patterns

The median time from treatment to last follow-up was 20 months (6 to 35 months). The 2-year Kaplan-Meier cumulative incidence of LR control and OS were 76.5% (95% confidence interval [CI]: 69-89%) and 95% (95% CI: 90-100%), respectively. Of the 16 patients who presented disease-related events, 13 exhibited LR failures and three had distant metastasis without LR failure. Of the 13 patients who developed LR failures, four displayed isolated local failure, four isolated regional failure, two local and regional failures, and the remaining three simultaneous LR and distant relapses. The clinical and dosimetric characteristics of the 13 LR failure patients are summarized in Table 2.

**Table 2:** Characteristics and dose volume data for patients with a locoregional failure

**No.**	**Primary site**	**T stage**	**N stage**	**V95 of the PTV**	**Location of the *Vf***	*Vf*-V95* (site of failure)	**Time to recurrence (months)**
1	Oropharynx	T3	N2b	95%	In-field	99% (N)	8
2	Larynx	T2	N2c	93%	In-field	99% (N)	persistence
3	Oral cavity	T4	N2c	92%	In-field	99% (T)	persistence
4	Larynx	T3	N2c	95%	Marginal	50% (T)	9
5	Oropharynx	T4	N3	97%	In-field	100% (T + N)	7
6	Oral cavity	T4	N0	98%	In-field	99% (T)	16
7	Hypopharynx	T3	N2c	96%	In-field	94% (N)	15
8	Oropharynx	T3	N2b	89%	In-field	100% (N)	15
9	Oropharynx	T3	N1	96%	In-field	100% (N)	persistence
10	Oral cavity	T3	N2a	96%	In-field	95% (T + N)	10
11	Larynx	T2	N2b	95%	In-field	100% (T) 100% (N)	persistence
12	Oropharynx	T3	N2b	97%	In-field	99% (T)	8
13	Hypopharynx	T3	N2b	99%	In-field	100% (T + N)	9

Abbreviations: *Vf *failure volume; *T *Tumor; *N *Node, * Percentage of *Vf *covered by 95% of the prescription dose.

Almost all recurrences occurred in previous disease areas. Only 1 of the 13 LR relapse patients exhibited a marginal failure, with the remaining 12 presenting with truly in-field failure within the high-dose region (PTV66-70). None of the study cohort patients had out-of-field LR failure. In the marginal failure patient, local recurrence developed in the primary site initially covered by 95% of 70Gy isodose. There was only one recurrence in the contralateral neck. This patient with persistent T2N2bM0 laryngeal squamous cell carcinoma underwent total laryngectomy with bilateral neck dissection. The final pathology report confirmed LR disease persistence, with metastatic disease in a solitary small node involving contralateral Level IV nodal region considered at low-risk of subclinical disease (PTV56) in the RT treatment plan. In none of the patients relapse occurred in the vicinity of the spared parotid gland or SMG, or in the spared OC.

Surgical salvage for LR failure (either persistent or recurrent disease) was attempted in five patients.

### Salivary gland-sparing

Contralateral parotid glands were spared in 98% of patients, and ipsilateral parotids in 54%. Concerning SMG, 18 (26%) contralateral glands were spared, and ipsilateral SMG were deemed to have been spared in only five. In these patients, ipsilateral Levels II and III were considered at low-risk, and the RT dose was 54-56Gy. In the other 13 (19%) patients, doses to the SMG were below 50Gy. The OC was spared to a dose ≤40Gy in 26 patients (37%), and to a dose <50Gy in another 30% (21 patients). Dosimetric data is detailed in Table 3.

**Table 3:** Mean doses to the spared salivary glands and oral cavity by primary tumor site

**Tumor site**	**Patients (nb)**	**Ipsilateral PG**		**Contralateral PG**		**Ipsilateral SMG**		**Contralateral SMG**		**OC**	
		**No. of spared glands**	**Mean dose Gy (range)**	**No. of spared glands**	**Mean dose Gy (range)**	**No. of spared glands**	**Mean dose Gy (range)**	**No. of spared glands**	**Mean dose Gy (range)**	**Nb**	**Mean dose Gy (range)**
Oral cavity	8	5	31 (29.4-32.5)	8	27.1 (16–31.7)	NS	NA	NS	NA	NS	NA
Oropharynx	31	12	28.6 (25–31.5)	30	27.5 (23.6-32.2)	2	35.5 (33–38)	8	33.8 (29–36.5)	5	34.2 (29.7-38.2)
Hypopharynx	17	9	28.9 (24.4-31.5)	17	27.6 (22.9-34.4)	1	39.6	4	36.1 (28.7-39.5)	9	34.8 (30.5-40)
Larynx	10	9	26.8 (20.7-33.2)	10	25 (19.1-30.1)	1	34.9	3	36.7 (34–39)	9	30.5 (23–39.7)
Nasopharynx	3	2	30.2 (30.4-30)	3	27.8 (26.7-28.7)	1	38	2	33.1 (30.3-35.8)	3	33.5 (29.7-35.2)
Unknown primary	1	1	32.5	1	17.3	NS	NA	1	28	1	44.3

Abbreviations: *PG *parotid gland; *SMG *submandibular gland; *OC* oral cavity; *NS *Non spared; *NA *Non applicable.

Xerostomia was assessed according to radiation therapy oncology group (RTOG) criteria [16]. At the last follow-up visit, none of the patients had developed Grade 3 permanent xerostomia, and 18.4% presented Grade 2 xerostomia.

### Correlation test and impact of dose parameters on local recurrence risk

In LR failure patients, *Vf*-V95 did not significantly correlate with the mean dose in both the ipsilateral and contralateral salivary glands (PG, SMG, and OC). The primary cancer stage and N stage were the only parameters significantly increasing local failure risk (p = 0.04 and p = 0.03, respectively). The LR recurrence risk was increased neither by lower dose in the salivary glands nor by PTV coverage (V95).

## Discussion

This report aimed to analyze failure patterns in HNC patients treated with a comprehensive approach for salivary gland-sparing using definitive IMRT. With 2-year LR control rates of 76.5% and OS of 95%, the treatment outcomes presented in this report are consistent with those of other patient series treated with parotid-sparing RT [15,17,18]. The recently-published randomized PARSPORT trial has proved that both objective and subjective xerostomia was significantly improved when parotid gland-sparing IMRT was compared with conventional RT [3]. However, there was concern about the higher number of locoregional recurrences in the IMRT arm (12 patients in the IMRT arm *versus* seven in the conventional arm). Though this difference was not statistically significant, some questions have been raised regarding treatment reproducibility in different centers, evaluation methodology for locoregional tumor extension, as well as delivery accuracy of IMRT. As part of quality control practices, individual center evaluation appears appropriate when a new treatment approach is adopted. On the whole, the number of marginal recurrences published in scientific literature was around 1.5%, depending on the series [19]. In our study, almost all recurrences occurred in-field in the high-dose volume region, and there was only one marginal recurrence. In none of the patients did recurrence occur in the vicinity of the parotid glands, SMG, or OC that were supposed to be spared. Even if our series comprised a relatively limited number of recurrences, this in-field high-dose recurrence rate data allows us to conclude that local control was not compromised by SMG, OC, and parotid gland-sparing strategy. Patterns of failure may depend also on the primary tumor sites thus an increase in the number of patients by tumor sites would have refined the results. A high Evidence-Based Medicine level would have however imply a non-inferiority design of the study.

Scarce data has been published concerning the safety of both SMG and OC preservation. A series published by Saarilahti et al. [8], and recently updated by Collan et al. [9] involving 80 patients treated using definitive RT (49%) and post-operative RT (51%) with the objective of parotid gland and SMG-sparing, showed no recurrences in the vicinity of spared structures, with mean doses ≤33.6Gy to SMG. Achieving such dose levels without underdosing the neighboring PTV is a challenge, particularly in patients with locally-advanced disease (77% in our study) who are unsuitable for surgical therapy. Without a doubt, the most reliable prognostic information is obtained from the pathology report when surgery is the primary management method. In the postoperative setting, dose levels are accurately adapted depending on the risk level. Given this context, other options for SMG-sparing have been explored. For instance, SMG transfer prior to RT could be an option for patients scheduled to undergo primary surgery with low-risk of contralateral lymph node Level IB involvement. This surgical technique has recently proven to be reproducible in a multicenter setting [20,21].

The key role of SMG and minor salivary glands in the production of unstimulated saliva and in subjective sensations of xerostomia is today well-recognized [5,22]. Dose levels administered to SMG and minor salivary glands represented by the OC were identified to be significant predictors of patient-reported xerostomia ^5^. More recently, Little et al. [22] showed xerostomia to be favorably affected by reducing the dose administered to all salivary glands other than parotid. Low patient-reported and observer-graded xerostomia was observed if the contralateral SMG mean dose was <50Gy and the OC mean dose <40Gy in patients whose bilateral parotid glands were spared as much as possible.

In our study involving mainly patients with locally-advanced disease, contralateral parotid glands were spared in 98% of patients, and ipsilateral parotid glands in 54%. Only 26% of contralateral SMG were spared to a dose <39Gy, because lymph node Level II was frequently considered at high-risk and treated to a dose of 60-63Gy. However, doses <50Gy were achieved in 51% of SMG. Achieving dose levels <50Gy to SMG when radical RT is used as primary therapeutic modality is presumed more realistic than <39Gy. In addition, we were able to keep mean doses to the OC <40Gy in 37% of patients. The SMG could not be spared mainly for OC primaries in our series, since they were not well lateralized and they were advanced stage. The majority of the small OC tumors are usually treated by surgery. In relation to OC-sparing, it should be noted that a substantial proportion of patients (44%) presented with oropharyngeal primary tumors and 11% with oral cavity primary tumors, which exposed this structure to high-dose levels.

As for xerostomia, although our retrospective study has its limitations, we would like to highlight that only 18.4% of our patients had Grade 2 xerostomia, with none exhibiting Grade 3 xerostomia. These results are considerably more favorable than those of another randomized study reporting Grade ≥2 xerostomia in 38% [3]. However, prospective trials must be undertaken to assess the final impact on patient quality of life.

Taking all the above points into consideration, it seems reasonable to maintaining mean doses of RT to the OC and SMGs as low as possible, in addition to parotid glands. Yet, for this approach, patients must be carefully selected. Sanguineti et al. [12] identified a non-significant risk (<5%) of Level IB involvement, even in patients with multiple positive neck levels suffering from early-stage oropharyngeal carcinomas. Eisbruch et al. [23] recommended that in nasopharyngeal, oropharyngeal or laryngeal cancer cases, selective Level IB-sparing could be considered if Level II is not grossly involved. Having followed these recommendations in our clinical practice, our study findings revealed that a comprehensive approach for salivary gland-sparing in definitive IMRT may constitute a safe strategy. Yet the safety of this approach must be confirmed by other larger-scale prospective studies.

## Conclusion

Locoregional control does not appear to be compromised by SMG and OC-sparing, in addition to parotid gland-sparing, in HNC patients treated using definitive IMRT. Most LR recurrences were in-field and located in regions covered by 95% of the prescription dose. As our series does not reveal a higher recurrence rate, our strategy consisting of sparing not only the parotid glands but also SMG and OC appears to be safe.

## Competing interest

All authors declare no conflict of interest.

## Authors’ contributions

All authors were responsible for patients treatments and care. EC and RDC wrote the manuscript. EC collected the patients’ data. RDC performed all statistcal analyses. All authors helped, read and approved the final manuscript.
